# What the papers say

**DOI:** 10.1093/jhps/hnx044

**Published:** 2017-12-06

**Authors:** Ajay Malviya

The *Journal of Hip Preservation Surgery* (*JHPS*) is not the only place where work in the field of hip preservation may be published. Although our aim is to offer the best of the best, we continue to be fascinated by work that finds its way into journals other than our own. There is much to learn from it so *JHPS* has selected six recent and topical subjects for those who seek a summary of what is taking place in our ever-fascinating world of hip preservation. What you see here are the mildly edited abstracts of the original articles, to give them what *JHPS* hopes is a more readable feel. If you are pushed for time, what follows should take you no more than 10 min to read. So here goes … 

## DOES THE CHONDRAL FLAP COMMONLY NOTED DURING HIP ARTHROSCOPY HAVE VIABLE TISSUE?

A chondral flap is a common finding during impingement surgery and it is a dilemma as to how this should be managed. The choice between preservation and reattachment in contrast to debridement and microfracture would depend on individual philosophy and of course the potential viability of the chondral flap that can serve as tissue encouraging the healing process. Two studies have recently looked at the viability of chondral flaps, with interestingly contrasting findings. 

Wright *et al*. [1] in a multi-centred collaboration proposed that full-thickness flaps may have viable chondrocytes residing in the cartilage flap despite shear trauma. In a laboratory-based study they set out to determine the *in vivo* tissue viability of acetabular chondral flaps in patients with femoroacetabular impingement (FAI) when samples were analysed immediately after biopsy. They hypothesized that the majority of the tissue in acetabular chondral flaps is viable in the joint microenvironment. Partially detached cartilage flaps from 10 patients undergoing arthroscopic hip surgery for FAI were biopsied in a minimally traumatic manner before chondroplasty and microfracture. Samples were placed in cold Hank’s Balanced Salt Solution without phenol red solution and immediately transported on ice to the laboratory. The edge of the samples was trimmed and further cut into three separate, 1-mm-thick sections. Sections were stained using a live/dead staining kit. Images were obtained with confocal microscopy, and the percentage of live cells was quantified. Patients averaged 36 ± 11 years (range 18–48 years), and 2 patients were female. The mean body mass index (BMI) was 28.9 ± 5.6 kg/m^2^. The total proportion of live cells from all sections analysed was 85.8%. The study determined that acetabular chondral flaps are ∼87% live cells when analysed immediately after biopsy, with 6 of the 10 patients having >90% live cells. These data point to the importance of laboratory techniques in making viability judgments in biological systems. They concluded that identification of >85% chondrocyte viability supports a foundation for evaluation and creation of novel clinical innovations for repair and replacement techniques using the flap as donor tissue, as alternatives to chondroplasty and microfracture.

Rodriguez-Fontan *et al*. [2] also set out with similar objectives. They hypothesized that chondral flaps from patients with cam lesions of the hip would exhibit less viability and greater tissue degeneration than would those of a matched control group. Patients with cam-type FAI who were treated with hip arthroscopy between 2014 and 2016 were asked to participate in this study. The cartilage lesions were localized and classified intraoperatively according to Beck classification. A chondral flap (study group) and a cartilage sample (control group) were obtained from each patient for histological evaluation. Cellular viability and tissue quality were examined and compared in both groups. Cellular viability was determined with live/dead staining, and tissue quality was evaluated using safranin O/fast green, haematoxylin and eosin (H&E) staining and immunohistochemistry for Collagen II. The Osteoarthritis Research Society International (OARSI) grading was used for quality assessment, and Image J software was used to calculate the percentage of tissue viability and Col II stain. A total of 10 male patients with a mean age of 38.4 years (range 30–55 years) were enrolled. All chondral flaps were classified as Beck grade 4. The mean cellular viability of the chondral flaps was reduced (54.6 ± 25.6%), and they were found to be degenerated (OARSI grade 4 ± 1.27). Control samples also had reduced viability (38.8 ± 30.3%) and were degenerative (OARSI grade, 3.5 ± 1.38). There was no statistically significant intergroup difference for viability (*P* = 0.203) or OARSI grade (*P* = 0.645), nor was there an intragroup correlation between viability and OARSI grade (*P* > 0.05). A significant negative correlation (*r* = −0.9, *P* = 0.035) was found between OARSI grade and Cllagen II percentage scale in five selected samples. The authors concluded that despite appearing normal macroscopically, the chondral flaps from patients with cam-type FAI displayed loss of viability and tissue degeneration. In addition, control samples obtained away from the injury area also displayed cartilage damage and degeneration. Careful consideration should be taken when attempting to reattach the chondral flap.

Both the studies have contrasting findings, but indicate the inherent problems with conclusions based on small sample size and laboratory-based research where technique of isolation, transfer of tissue, time to assess the specimens, and indeed tissue handling are critical. For the time being there remains an equipoise and perhaps we need further studies to clarify the issue.

## DO PATIENTS UNDERGOING HIP ARTHROSCOPY REQUIRE CHEMICAL PROPHYLAXIS FOR VENOUS THROMBOEMBOLISM?

Haldane *et al*. [3] from McMaster University, Hamilton, Canada performed a systematic review on hip arthroscopy with a focus to report the venous thromboembolism (VTE) incidence in patients who receive VTE prophylaxis and those who do not; report how VTE prophylaxis is currently being administered; and report operative and patient-related risk factors for VTE identified in the literature.

Data were collected regarding VTE prophylaxis, traction use, surgical time, VTE incidence, patient and operative factors, and postoperative weight bearing and rehabilitation. Study quality was assessed in duplicate with the Methodological Index for Non-Randomized Studies criteria. The outcome analyses included 14 studies that involved 2850 patients (2985 hips). The weighted mean follow-up period was 19 ± 8 months, ranging from 7 days to 103 months. The weighted mean age was 40.7 ± 7 years, ranging from 6 to 82 years, and 39.6% of patients were male patients. The overall weighted proportion of VTE events after hip arthroscopy found in 14 included studies was 2.0%, with 25 VTE events. Several studies reported patient risk factors, which included increased age, increased BMI, prolonged traction time and use of oral contraceptives.

The use and efficacy of VTE prophylaxis are highly under-reported within hip arthroscopy. The low incidence of VTE events found in this review (2.0%) suggests that prophylaxis may not be necessary in low-risk patients undergoing hip arthroscopy; however, the true rate may be under-reported. Current literature suggests that prophylaxis is typically not prescribed. Early mobility and postoperative rehabilitation may also help to further mitigate the risk of VTE events, but use of these strategies needs further prospective evaluation.

## DOES LABRAL REATTACHMENT IN (FAI) SURGERY RESULT IN INCREASED 10-YEAR SURVIVORSHIP COMPARED WITH RESECTION?

Since the importance of an intact labrum for normal hip function has been shown, labral reattachment has become the standard method for open or arthroscopic treatment of hips with FAI. However, no long-term clinical results exist evaluating the effect of labral reattachment.

Anwander *et al*. [4] looked at the 10-year results of a previous study comparing open surgical treatment of FAI with labral resection versus reattachment performed at Bern University Hospital, Switzerland. Between June 1999 and July 2002, they performed surgical hip dislocation with femoral neck osteoplasty and acetabular rim trimming in 52 patients (60 hips) with mixed-type FAI. In the first 20 patients (25 hips) until June 2001, a torn labrum or a detached labrum in the area of acetabular rim resection was resected. In the next 32 patients (35 hips), reattachment of the labrum was performed. The same indications were used to perform both procedures during the periods in question. Of the 20 patients (25 hips) in the first group, 19 patients (95%) (24 hips [96%]) were available for clinical and/or radiographical follow-up at a minimum of 10 years (mean 13 years; range 12–14 years). Of the 32 patients (35 hips) in the second group, 29 patients (91%) (32 hips [91%]) were available for clinical and/or radiographical follow-up at a minimum of 10 years (mean 12 years). The anterior impingement test was used to assess pain. Function was assessed using the Merle d'Aubigné-Postel score and ROM. Survivorship calculation was performed using the method of Kaplan–Meier with failure defined as conversion to total hip arthroplasty (THA), progression of osteoarthritis (OA) (of one grade or more on the Tönnis score), and a Merle d'Aubigné-Postel score <15.

At the 10-year follow-up, hip pain in hips with labral reattachment was slightly improved for the postoperative Merle d'Aubigné-Postel pain subscore (*P* = 0.017). No difference existed for the prevalence of hip pain assessed using the anterior impingement test with the numbers available (*P* = 0.062). Function was slightly better in the reattachment group for the overall Merle d'Aubigné-Postel score (*P* = 0.028) and hip abduction (*P* = 0.001). Hips with labral reattachment showed a better survival rate at 10 years than did hips that underwent labral resection (78% versus 46% *P* = 0.009) with the endpoints defined as conversion to THA, progression of OA, and a Merle d'Aubigné-Postel score <15. With isolated endpoints, survival at 10 years was increased for labral reattachment and the endpoint Merle d'Aubigné score <15 (*P* = 0.009) but did not differ for progression of OA (*P* = 0.957) or conversion to THA (*P* = 0.366).

The evidence reported in this study suggests the importance of preserving the labrum and shows that resection may put the hip at risk for early deterioration. At 10-year follow-up, hips with labral reattachment less frequently had a decreased Merle d'Aubigné score, however, no effect on progression of OA or conversion to THA could be shown.

## DOES PARTICIPATION IN SPORTS AFFECT OSTEOARTHRITIC PROGRESSION AFTER PERIACETABULAR OSTEOTOMY?

Hara *et al.* [5] from Fukuoka, Japan wished to explore whether postoperative participation in sports leads to progression of the Kellgren–Lawrence (KL) grade of OA after periacetabular osteotomy (PAO) for hip dysplasia.

The authors retrospectively reviewed data on 161 patients (183 hips) who underwent PAO for symptomatic acetabular dysplasia with preoperative KL grade 1 or 2 between 1998 and 2011. The mean age at the time of surgery was 42.0 years (range 12–64 years), and the mean follow-up duration was 100 months (range 13–180 months). Data included participation in sports, the University of California, Los Angeles (UCLA) activity scale score, age at the time of surgery, BMI, follow-up duration, history of treatment for developmental hip dislocations, Merle d'Aubigné-Postel score, Oxford Hip Score, centre-edge angle and KL grade. Univariate and multivariate analyses were applied to determine which factors were associated with progression to KL grade 3 or 4 after PAO.

The number of patients who participated in sports significantly increased from 50 (31.1%) preoperatively to 89 (55.3%) postoperatively. The mean UCLA score significantly increased from 4.7 preoperatively to 5.5 postoperatively. The KL grade progressed to grade 3 or 4 in 16 hips, including 4 hips that underwent conversion to THA. No significant differences were found in postoperative participation in sports (89 hips [53.3%] versus 11 hips [68.8%], respectively; *P* = 0.24) and the UCLA score (*P* = 0.30) between hips with KL grade 1 or 2 and KL grade 3 or 4. A multivariate analysis revealed that no factors, including postoperative participation in sports, were significantly associated with progression to KL grade 3 or 4.

The authors concluded that postoperative participation in sports after PAO did not significantly and negatively influence progression of the KL grade at midterm follow-up.

## LONG-TERM SURVIVAL AFTER TRIPLE PELVIC OSTEOTOMY FOR ACETABULAR DYSPLASIA

Birmingham Interlocking Pelvic Osteotomy (BIPO) is a modification of triple osteotomy, developed in Birmingham, UK and is an alternative to the more commonly performed PAO for patients with hip dysplasia. Mei-Dan *et al*. [6] have looked at the long-term clinical and radiographical outcomes of BIPO in this prospective study. The study includes the clinical outcomes of the first 100 consecutive patients (116 hips; 88 in women, 28 in men) undergoing BIPO, reflecting the surgeon’s learning curve. Failure was defined as conversion to hip arthroplasty. The mean age at operation was 31 years (7–57). Three patients (three hips) were lost to follow-up.

Survivorship was 76% at 10 years and 57% at a mean of 17 years. Younger patients (<20 years) had the best survivorship (20 hips at risk; 90% at 17 years; 95% confidence interval 65–97). Post-operative complications occurred after 12 operations (10.4%) over the duration of the study. Increasing patient age and hip arthritis grade were primary determinants of surgical failure.

BIPO provides good-to-excellent survivorship in appropriately selected patients, with a relatively low rate of complications. The authors suggest that their results are comparable with other established methods of PAO, such as the Bernese PAO, even during the surgeon’s initial learning curve. It can be an alternative to the more commonly performed PAO for this group of patients.

## HOW COMMON IS DELAYED UNION AFTER PERI-ACETABULAR OSTEOTOMY (PAO)?

Delayed union is a concern after pelvic osteotomies in adults and may have a bearing in recovery of patients. Akiho *et al*. [7] from Fukuoka University Faculty of Medicine, Japan looked at the incidence of delayed union one year after PAO using X-ray and CT scans.

This study is a retrospective review of 150 hips in 132 consecutive patients with acetabular dysplasia who underwent PAO between January 2012 and June 2016 and evaluated 107 hips for which pelvic CT scans taken at 1 year after PAO were available. Clinical evaluations included age at surgery, weight, BMI and history. Radiographical evaluations were to assess pubic, ischial and iliac delayed union at 1 year post-operatively.

Based on X-ray analysis, the incidence of delayed union in the pubic, ischial and iliac bones was 11.2% (12 hips), 5.6% (6 hips) and 0% (0 hips), respectively, and 20.6% (22 hips), 8.4% (9 hips) and 0% (0 hips), respectively, based on CT scans.

The authors concluded that the incidence of delayed union of the pubis and ischium at 1 year after PAO according to CT scans was higher than that based on X-ray imaging. CT scans are useful in patients with some symptoms at the osteotomy site.
